# Investigating Biological Activity Spectrum for Novel Styrylquinazoline Analogues

**DOI:** 10.3390/molecules14104246

**Published:** 2009-10-23

**Authors:** Josef Jampilek, Robert Musiol, Jacek Finster, Matus Pesko, James Carroll, Katarina Kralova, Marcela Vejsova, Jim O'Mahony, Aidan Coffey, Jiri Dohnal, Jaroslaw Polanski

**Affiliations:** 1 Zentiva k.s., U kabelovny 130, 102 37 Prague 10, Czech Republic; E-Mail: jiri.dohnal@zentiva.cz (J.D.); 2 Department of Chemical Drugs, Faculty of Pharmacy, University of Veterinary and Pharmaceutical Sciences, Palackeho 1-3, 612 42 Brno, Czech Republic; 3 Institute of Chemistry, University of Silesia, Szkolna 9, 40007 Katowice, Poland; E-Mails: robert.musiol@us.edu.pl (R.M.); jfinster@us.edu.pl (J.F.); polanski@us.edu.pl (J.P.); 4 Department of Ecosozology and Physiotactics, Faculty of Natural Sciences, Comenius University, Mlynska dolina Ch-2, 84215 Bratislava, Slovakia; E-Mail: matus.pesko@gmail.com (M.P.); 5 Department of Biological Sciences, Cork Institute of Technology, Bishopstown, Cork, Ireland; E-Mails: james.carroll@cit.ie (J.C.); jim.omahony@cit.ie (J.M.); aidan.coffey@cit.ie (A.C.); 6 Institute of Chemistry, Faculty of Natural Sciences, Comenius University, Mlynska dolina Ch-2, 84215 Bratislava, Slovakia; E-Mail: kralova@fns.uniba.sk (K.K.); 7 Department of Biological and Medical Sciences, Faculty of Pharmacy in Hradec Kralove, Charles University in Prague, Heyrovskeho 1203, 500 05 Hradec Kralove, Czech Republic; E-Mail: marcela.vejsova@faf.cuni.cz (M.V.)

**Keywords:** styrylquinazolinone and styrylquinazoline derivatives, lipophilicity, PET inhibition, spinach chloroplasts, *in vitro* antimycobacterial activity, *in vitro* antifungal activity, structure-activity relationships

## Abstract

In this study, series of ring-substituted 2-styrylquinazolin-4(3*H*)-one and 4-chloro-2-styrylquinazoline derivatives were prepared. The syntheses of the discussed compounds are presented. The compounds were analyzed by RP-HPLC to determine lipophilicity. They were tested for their inhibitory activity on photosynthetic electron transport (PET) in spinach (*Spinacia oleracea* L.) chloroplasts. Primary *in vitro* screening of the synthesized compounds was also performed against four mycobacterial strains and against eight fungal strains. Several compounds showed biological activity comparable with or higher than that of the standard isoniazid. It was found that the electronic properties of the R substituent, and not the total lipophilicity of the compound, were decisive for the photosynthesis-inhibiting activity of tested compounds.

## 1. Introduction

A quinoline moiety is present in many classes of biologically-active compounds. A number of them have been clinically used as antifungal, antibacterial and antiprotozoic drugs [1,2], as well as antituberculotic agents [3,4,5]. Some quinoline-based compounds have also shown antineoplastic, antiasthmatic and antiplatelet activity [6,7,8,9,10,11]. A series of compounds derived from 8-hydroxyquinoline and styrylquinoline derivatives were recently synthesized as potential HIV-1 integrase inhibitors [12,13,14,15]. Our previous study dealing with 8-hydroxyquinoline and styrylquinoline derivatives showed that they could also possess strong antifungal activity [16,17]. According to recently reported results, some new hydroxyquinoline derivatives also possess interesting herbicidal activities [16,18,19,20]. In addition, some of the investigated quinoline derivatives also showed antineoplastic activity [18,21].

Over 50% of commercially available herbicides act by reversibly binding to photosystem II (PS II), a membrane-protein complex in the thylakoid membranes which catalyses the oxidation of water and the reduction of plastoquinone [22] and thereby inhibit photosynthesis [23,24,25]. Some organic compounds, e.g. substituted benzanilides [26] or substituted anilides of 2,6-disubstituted pyridine-4-thiocarboxamides [27] or pyrazine-2-carboxylic acids [28,29] were found to interact with tyrosine radicals TyrZ and TyrD which are situated in D1 and D2 proteins on the donor side of PS II. Due to this interaction interruption of the photosynthetic electron transport occurred.

Tuberculosis (TB) is a worldwide pandemic. About 1/3 of the world's population is infected with *Mycobacterium tuberculosis*, and almost two million people die every year as a result. A large number of infected people are carriers of the latent form, which creates a potentially dangerous future source of the illness. The HIV pandemic has also led to the rapid growth of the TB epidemic, and increased the likelihood of people dying of TB. Another factor contributing to the rise in TB infections, and consequently to the increased number of deaths, is the appearance of multiple drug-resistance (MDR), *i.e.*, rise of multidrug-resistant TB (MDR-TB) [30,31].

The *Mycobacterium* genus is composed of the *M. tuberculosis* complex and other species known as nontuberculous mycobacteria (NTM, or MOTT – mycobacteria other than tuberculosis). In recent decades, the decrease in the prevalence of tuberculosis in developed countries has resulted in an increase in the proportion of diseases caused by NTM [32]. Among these species, the *M. avium* complex (MAC) has emerged as a major human pathogen, being a common cause of disseminated disease and death in patients with HIV/AIDS [33].

Chronic pulmonary disease is the most common clinical manifestation among the diseases caused by NTM, and the most common pathogens are the species belonging to the MAC, followed by *M. kansasii*. The clinical characteristics of NTM-related pulmonary disease are, in many cases, extremely similar to those of tuberculosis. Other clinical manifestations are caused principally by *M. fortuitum*, *M. smegmatis* and *M. abscessus* due to peritoneal infection as a result of catheterization or postsurgical infections [34]. The above mentioned non-tuberculous strains are sometimes resistant to commonly used drugs (isoniazid, rifampicin, pyrazinamide and ethambutol) and other antituberculous drugs [30]. Therefore, systematic development of new effective compounds is necessary. Similarly, there is also an urgent need for discovery of new drugs with novel modes of action for the treatment of systemic mycoses. This is due to the rapid growth of the immunocompromised patient population and development of resistance to current azole therapies, and the high toxicity of polyenes [35]. It should be stressed that hydroxyquinolines and their derivatives were introduced as antifungal or antimycobacterial agents in clinical practice and novel compounds of this type are still being investigated [3,4,5,36,37].

This is a follow-up paper to our previous articles [12,13,14,15,16,17,18,19,20,21] dealing with synthesis and biological activities of ring-substituted quinazolinone derivatives. In the context of our previously-described azanaphtalenes, new modifications of quinoline moiety that can trigger interesting biological activity were investigated.

Primary *in vitro* screening of the synthesized compounds was performed against four mycobacterial strains and against eight fungal strains. The compounds were also tested for their photosynthesis-inhibiting activity (the inhibition of photosynthetic electron transport) in spinach chloroplasts (*Spinacia oleracea* L.). Lipophilicity (log *k*) of the compounds was determined using RP-HPLC. Relationships among the structure and *in vitro* antimicrobial activities or/and inhibitory activity related to inhibition of photosynthetic electron transport (PET) in spinach chloroplasts of the new compounds are discussed.

## 2. Results and Discussion

### 2.1. Chemistry

**Scheme 1:**
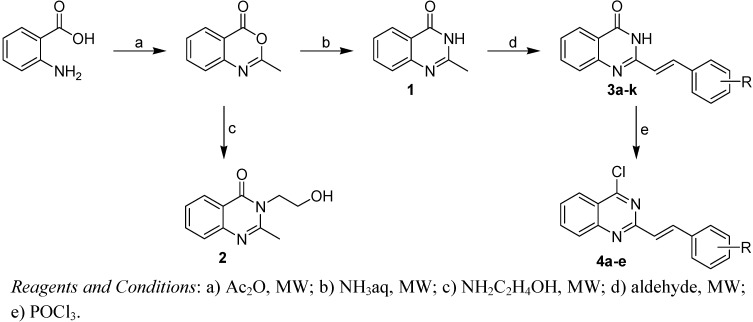
Synthetic pathway and general formula of prepared quinazolinone derivatives:

All the studied compounds were prepared according to Scheme 1. Microwave-assisted synthesis facilitated the preparation of quinazoline-related structures. 2-Methyl-4*H*-benzo[*d*][1,3]oxazin-4-one was synthesized from anthranilic acid and acetic anhydride. A further reaction with ammonia or hydroxylamine afforded 2-methylquinazolin-4(3*H*)-one (**1**) or 3-(2-hydroxyethyl)-2-methylquinazolin-4(3*H*)-one (**2**). 2-Styrylquinazolin-4(3*H*)-ones **3a**-**k** were obtained from appropriate aldehydes using neat microwave-assisted synthesis [38]. Further chlorination/dehydratation with POCl_3_ yielded 4-chloro-2-styrylquinazoline derivatives **4a**-**e**. Styrylquinazolines can exist as *E-* or *Z-*isomers according to the orientation of the ethylene linker. This can greatly affect their biological activity *i.e.*, behaviour at the site of action and complexation mechanism(s). Thus we have studied the isomerism of all the obtained compounds with NMR techniques and crystallography, as formerly described for similar structures [39,40]. Fortunately this feature can be easily determined by examination of the coupling constants of both vinyl protons in the spectra. These are much higher in the case of *E-*isomers (*J* > 16 Hz) compared to *Z-*isomers (*J* < 12 Hz) [39]. All styrylquinazolines were found to be pure *E-*isomers, which is in good agreement with previous results [13,17,18,39,40].

### 2.2. Lipophilicity

Many low molecular weight drugs cross biological membranes through passive transport, which strongly depends on their lipophilicity. Lipophilicity is a property that has a major effect on absorption, distribution, metabolism, excretion, and toxicity (ADME/Tox) properties, as well as pharmacological activity. Lipophilicity has been studied and applied as an important drug property for decades [41].

This thermodynamic parameter describes the partitioning of a compound between aqueous and organic phases and is characterized by the partition (log *P*) coefficient [42,43]. With new computerized methods of log *P* calculation, the possibility of predicting hydrophobicity in large libraries of compounds came into being. Lipophilicity computing software can usually calculate log *P* and Clog *P*. The software calculates log *P* values as lipophilicity contributions/increments of individual atoms, fragments and the pairs of interacting fragments in the chemical structure, *i.e.*, increments of carbon and hetero atoms, aromatic systems and functional groups. The software calculates lipophilicity contributions according to different internal databases/libraries, so the calculated lipophilicity values are dependent on the software used, and the values for individual compounds may be different. This fact, as well as various ionic/zwitterionic forms and intramolecular interactions, may cause differences between calculated and experimentally determined lipophilicities.

Classical methods for determination of these partition constants are time consuming and not always adequately reliable. It was recognised some time ago that the retention of a compound in reversed-phase liquid chromatography is governed by its lipophilicity, and thus shows correlation with the octanol–water partition coefficient [44]. Reversed phase high performance liquid chromatography (RP-HPLC) provides an excellent platform for computer controlled automated measurements with computerised data acquisition for a large number of investigated compounds. Other advantages in the use of HPLC retention data for lipophilicity determination are the absence of need for concentration determination and method validation, simultaneous separation of small impurities from the main component, sufficiency of small amounts of material for measurements and possibility of their full automation. Therefore the investigation of the true potential of this method is of great importance [45].

The effect of stationary and mobile phase selection has been published by van der Waterbeemd *et al*. [43] and more recently by Claessens *et al*. [46]. RP-HPLC methods have become popular and widely used for lipophilicity measurement [47]. A general procedure is the measurement of the directly accessible retention time under isocratic conditions with varying amounts of methanol as an organic modifier in the mobile phase using end-capped non-polar C_18_ stationary RP columns and calculating the logarithm of capacity factors (log *k*). Log *k* is the logarithm of capacity factors in chromatographic approaches, which is related to the partitioning of a compound between a mobile and a (pseudo-)stationary phase. Log *k* is used as the lipophilicity index converted to log *P* scale [43,45,46,47,48,49].

Some groups have used a C_18_ chromatographic column with methanol-water mobile phases to obtain log *k*_w_, *i.e.*, the retention factor extrapolated to 0% organic modifier, as an alternative to log *P* [50]. The log *k*_w_ is obtained by performing several measurements with various ratios of water/organic solvent. Nevertheless determination of log *k*_w_ has some disadvantages in that it is time consuming due to the various measurements that need to be undertaken before calculation of log *k*_w_ [44]. The main reason for the measurement is that it is more convenient to perform a systematic study of log *k* of various heteroaromatic compounds using mobile phases containing around 50% methanol due to various intramolecular interactions between heteroatoms and substituents [51,52,53]. Therefore this study was performed using methanol/water (55:45) as the mobile phase. The conditions (non-buffered mobile phase) were chosen with respect to conditions of biological evaluations, which are performed mostly under neutral conditions (pH ~ 7). The lipophilicity data can be strongly influenced by intramolecular interactions under the applied chromatographic conditions which were investigated in the paper [54,55].

**Figure 1:**
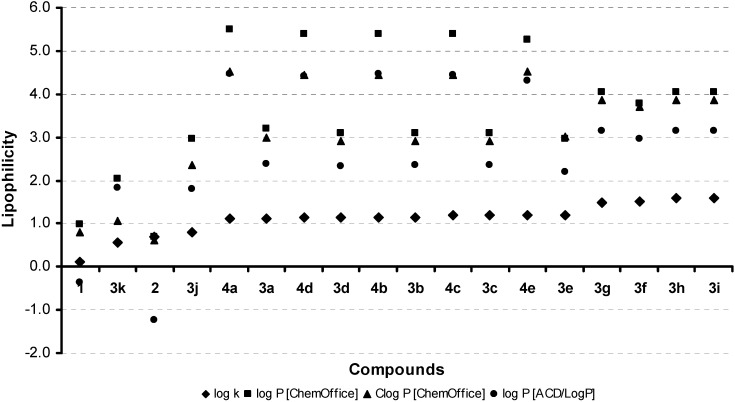
Comparison of the log *P*/Clog *P* values computed using the two programs with the calculated log *k* values. The discussed compounds **1**-**4e** are ordered according to the increase in log *k* values.

Lipophilicities (log *P*/Clog *P*) of all eighteen compounds **1**-**4e** were calculated using two commercially available programs (ChemDraw Ultra and ACD/LogP) and also measured by means of the RP-HPLC determination of capacity factors *k* with subsequent calculation of log *k*. The procedure was performed under isocratic conditions. Neither programme succeeded in resolving the differing lipophilicity values of individual positional isomers inasmuch as the same log *P*/Clog *P* values were calculated for **3b**-**d**, **3g**-**i** and **4b**-**d**. The results are shown in Table 1 and illustrated in Figure 1.

The results obtained with respect to all compounds show that the experimentally-determined lipophilicities (log *k* values) of all compounds are lower than those indicated by the calculated log *P*/Clog *P*, as shown in Figure 1, indicating that experimentally-determined log *k* values correlate relatively poorly with the calculated log *P*/Clog *P*. These facts are caused by both the above mentioned limitation of the software programmes used and also possibly to intramolecular interactions between heterocyclic nitrogens and substituents [19,54,55,56,57,58,59]. As expected, compound **3i** showed the highest lipophilicity, while compound **1** exhibited the lowest. Series **4a**-**e** showed lower lipophilicity (log *k*) than series **3a**-**e**, contrary to all calculated log *P*/Clog *P* data. The higher lipophilicity of **3a**-**e** compared with **4a**-**e** is caused by intramolecular interactions between N_(3)_ and carbonyl oxygen in the position 4. Similar interactions were described recently [19,55,56,57,58,59]. This hypothesis can be supported by the fact that log *k* of **1** was lower than **2**, although this is contrary to calculated log *P*/Clog *P*. When the lipophilicity of both compounds was calculated with intramolecular interactions between N_(3)_-H···O=C_(4)_, the lipophilicity increased (Clog *P* (**1**): 1.343 and Clog *P* (**2**): 2.2306). Similar facts may be observed at **3a**-**e** and **4a**-**e**, therefore the experimental log *k* data of **4a**-**e** were slightly lower than those log *k* of **3a**-**e**.

**Table 1:** Comparison of the calculated lipophilicities (log *P*/Clog *P*) with the determined log *k* values, electronic Hammett's parameters (σ) and bulk parameters MR (volume of substituents) [60].

**Comp.**	**R**	**log *k***	**log *P*/Clog *P*** ChemOffice	**log *P*** ACD/LogP	**σ** [60]	**MR** [60]
**1**	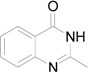	0.1170	0.98 / 0.804	-0.36 ± 0.59	–	–
**2**	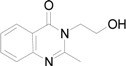	0.7047	0.69 / 0.6149	-1.23 ± 0.69	–	–
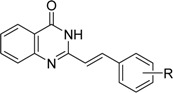						
**3a**	H	1.1148	3.21 / 2.997	2.37 ± 0.61	0.0	0.0
**3b**	2-OCH_3_	1.1509	3.09 / 2.916	2.35 ± 0.62	-0.390 [61]	6.5
**3c**	3-OCH_3_	1.1837	3.09 / 2.916	2.35 ± 0.62	0.115	6.5
**3d**	4-OCH_3_	1.1351	3.09 / 2.916	2.32 ± 0.62	-0.268	6.5
**3e**	2,4-OCH_3_	1.1982	2.96 / 3.005	2.20 ± 0.62	-0.658	13.0
**3f**	3-Cl	1.5059	3.77 / 3.710	2.96 ± 0.61	0.373	4.8
**3g**	2-Br	1.4830	4.04 / 3.860	3.14 ± 0.64	–	7.6
**3h**	3-Br	1.5904	4.04 / 3.860	3.14 ± 0.64	0.391	7.6
**3i**	4-Br	1.5927	4.04 / 3.860	3.14 ± 0.64	0.232	7.6
**3j**	4-CHO	0.8005	2.96 / 2.350	1.79 ± 0.63	1.030	5.3
**3k**	2,3,4-OH	0.5510	2.05 / 1.066	1.84 ± 0.63	–	4.5
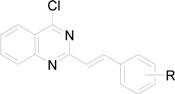						
**4a**	H	1.1088	5.50 / 4.51522	4.47 ± 0.56	0.0	0.0
**4b**	2-OCH_3_	1.1497	5.38 / 4.43422	4.47 ± 0.57	-0.390 [61]	6.5
**4c**	3-OCH_3_	1.1827	5.38 / 4.43422	4.44 ± 0.57	0.115	6.5
**4d**	4-OCH_3_	1.1337	5.38 / 4.43422	4.41 ± 0.57	-0.268	6.5
**4e**	2,4-OCH_3_	1.1953	5.25 / 4.52322	4.30 ± 0.57	-0.658	13.0

The presence of phenolic and carbonyl moieties decreased the lipophilicity. Nevertheless, the observation that the lipophilicity of compounds **3k** and **3j** was close to the lipophilicity of compound **2** was unexpected. Compound **3f** showed less lipophilicity compared with **3h**. On the basis of comparison of the lipophilicity data log *k* of both Br-substituted isomers **3g**-**3i**, it can be stated that 4-bromo derivative **3i** possessed higher lipophilicity than the 3-bromo isomer **3h** and the 2-bromo isomer **3g**. A diverse trend can be observed at methoxy moiety substituted compounds **3b**-**e** and **4b**-**e**. Compounds **3e** and **4e** showed the highest lipophilicity, while compounds **3a** and **4a** possessed the lowest lipophilicity within individual series of methoxy moiety substituted compounds, according to log *k* data. Compounds **3a** and **4a** showed lower lipophilicity in comparison with the results calculated by the software. If the lipophilicity data log *k* of three position isomers **3b**-**d**, **4b**-**d** are compared, it can be stated that 3-methoxy derivative **3c**/**4c** possessed higher lipophilicity than 2-methoxy derivative **3b**/**4b** and 4-methoxy derivative **3d**/**4d** showed the lowest lipophilicity.

Generally, based on the facts discussed above, it can be stated that intramolecular interactions, especially within styrylquinazolinone derivatives, play a significant role in the lipophilicity of the discussed compounds. It can be assumed that the determined log *k* data specify lipophilicity within the individual series of compounds. Lipophilicity increased in the following order: 2,3,4-OH < 4-CHO < H < 4-OCH_3_ < 2-OCH_3_ < 3-OCH_3_ < 2,4-OCH_3_ < 2-Br < 3-Cl < 3-Br < 4-Br.

### 2.3. Inhibition of photosynthetic electron transport (PET) in spinach chloroplasts

The evaluated quinazoline derivatives showed relatively low activity related to inhibition of photosynthetic electron transport (PET) in spinach chloroplasts (Table 2). Compounds **4a** and **4c** expressed the highest PET-inhibiting activity (IC_50_: 285 and 303 µmol/L, respectively). PET inhibition by several compounds (**1**, **3a**-**c**, **3g**, **3j** and **4b**) could not be determined due to precipitation of the compounds during the experiment and compound **3k** interacted with the artificial electron acceptor DCPIP (change of the colour). The PET-inhibiting activity was expressed by negative logarithm of IC_50_ value (compound concentration in mol/L causing 50% inhibition of PET). Despite the relatively low inhibitory activity of the studied compounds as well as the relative scarcity of compounds for which PET-inhibiting activity could be determined, the correlations between log (1/IC_50_) and log *k* or Hammett's parameters (σ) of the R substituent for both tested groups (**3d**-**i**, as well as **4a**-**d**) were performed. The σ values [60,61] mentioned in Table 1 were used for calculations; the σ value for R: 2,4-OCH_3_ was calculated from the sum of corresponding σ values for R: 2-OCH_3_ and R: 4-OCH_3_.

**Table 2:** IC_50_ values related to PET inhibition in spinach chloroplasts in comparison with 3-(3,4-dichlorophenyl)-1,1-dimethylurea (DCMU) standard and *in vitro* antimycobacterial activity MIC/IC_90_ of compounds **1**-**3i**, **4a**-**4e** in comparison with the standard, isoniazid (INH).

Comp.	PET inhibition IC_50_ [μmol/L]	MIC/IC_90_ [µg/mL]			
		*M. smegmatis*	*M. absessus*	*M. kansasii*	*M. avium* complex
**1**	^a^	>300	>300	>300	>300
**2**	362	>300	>300	>300	>300
**3a**	^a^	>300	>300	>300	>300
**3b**	^a^	>100	>100	>100	>100
**3c**	^a^	>100	>100	>100	>100
**3d**	693	>100	>100	>100	>100
**3e**	391	>100	80	20	80
**3f**	1034	>300	>300	>300	>300
**3g**	^a^	>100	>100	>100	>100
**3h**	561	>100	>100	>100	>100
**3i**	665	>100	>100	>100	>100
**4a**	285	>100	>100	>100	>100
**4b**	^a^	>100	80	60	80
**4c**	303	>100	>100	60	>100
**4d**	390	>100	>100	>100	>100
**4e**	508	>100	>100	>100	>100
**DCMU**	1.9	–	–	–	–
**INH**	–	39	>100	<10	<10

*^a^* precipitation during the experiment or interaction with DCPIP.

The importance of electronic properties of the R substituent was for the inhibitory activity (IC_50_ in mol/L) of compounds **3a**-**i** unambiguously much more significant than the compound lipophilicity (log *k*):

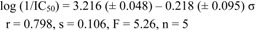
(1)

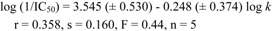
(2)


Similarly, the inhibitory activity (IC_50_ in mol/L) of compounds **4a**-**e** depended predominantly on the Hammett's constants (σ) of R substituents:

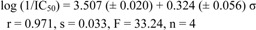
(3)

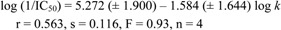
(4)


From Equations 1-4 it is evident that in both studied groups of compounds (**3d**-**i** and **4a**-**d**) the electronic properties of the R substituent were decisive for photosynthesis-inhibiting activity. For estimation of the potential contribution of the compound lipophilicity to its biological activity, a larger data set for both groups of compounds tested would be necessary.

### 2.4. In vitro antimycobacterial evaluation

Sixteen compounds **1**-**3i**, **4a**-**4e** were evaluated for their *in vitro* antimycobacterial activity against four mycobacterial strains and the results are shown in Table 2. According to the results, it is evident that the tested compounds were poorly soluble in the testing medium and therefore concentrations of the compounds in the medium were not sufficient for determination of real antimycobacterial activity. Due to this fact, it can be concluded that the majority of compounds evaluated did not show any significant antimycobacterial activity. Only 2-[(*E*)-2-(2,4-dimethoxyphenyl)vinyl]quinazolin-4(3*H*)-one (**3e**) and 4-chloro-2-[(*E*)-2-(2-methoxyphenyl)vinyl]quinazoline (**4b**) expressed an interesting MIC especially against *M. kansasii*, *M. avium* complex and *M. absessus*. Both compounds were more active than INH in case of *M. absessus*.

With the available data it is difficult to attempt to determine any structure-activity relationships, although some observations can be made. The 4-chloroquinazoline nucleus (series **4**) seems to be more advantageous for higher antimycobacterial activity than the quinazolin-4(3*H*)-one scaffold (series **3**); e.g., unsubstituted **3a** showed lower activity than **4a**.

The benzylidene part of the molecule is also important for antimycobacterial activity. Bulk parameters (the volume of substituents) MR [60] are also very important for activity. According to Table 1, Table 2 it can be assumed that compounds with bulky substituents showed higher antimycobacterial activity. Unsubstituted compounds **3a** and chloro substituted **3f** possessed less activity than methoxy or bromo substituted compounds. The highest effect was shown by disubstituted 2,4-methoxy derivative **3e**. The position of substituents on the benzylidene part of the molecule is important especially for compounds within series **4**; compare the activity of compounds **4b** > **4c** > **4d** > **4e**.

### 2.5. In vitro antifungal susceptibility testing

All quinazoline derivatives **1**-**4e** were tested for their *in vitro* antifungal activity against eight fungal strains. The antifungal activity of all the compounds were in the range from >125 to >500 μmol/L and therefore the activities are not presented in detail. According to these results, it can be concluded that all the compounds are almost completely insoluble in aqueous solvents as they precipitated from the testing medium. Generally, compounds **1** and **2** showed lower activity than most compounds **3** and **4**. The substitution of benzylidene part of the molecule by 3-OCH_3_ (**3c**, **4c**) or 2,3,4-OH (**3k**) seems to contribute to antifungal activity within both series of compounds.

## 3. Conclusions

Series of ring-substituted 2-styrylquinazolin-4(3*H*)-one and 4-chloro-2-styrylquinazoline derivatives were prepared and characterized. All eighteen prepared quinazoline derivatives were analyzed using a RP-HPLC method for lipophilicity measurement and their lipophilicity was determined. The prepared compounds were tested for their antifungal and antimycobacterial activity and for their activity related to the inhibition of photosynthetic electron transport (PET) in spinach chloroplasts (*Spinacia oleracea* L.). 2-[(*E*)-2-(2,4-Dimethoxyphenyl)vinyl]quinazolin-4(3*H*)-one (**3e**) and 4-chloro-2-[(*E*)-2-(2-methoxyphenyl)vinyl]quinazoline (**4b**) exhibited the highest *in vitro* antimycobacterial activity. 4-Chloro-2-[(*E*)-2-(3-methoxyphenyl)vinyl]quinazoline (**4c**) showed the highest PET-inhibiting activity.

## 4. Experimental

### 4.1. General

All reagents were purchased from Aldrich. Kieselgel 60, 0.040–0.063 mm (Merck, Darmstadt, Germany) was used for column chromatography. TLC experiments were performed on alumina-backed silica gel 40 F_254_ plates (Merck, Darmstadt, Germany). The plates were illuminated under UV (254 nm) and evaluated in iodine vapour. The melting points were determined on Boetius PHMK 05 (VEB Kombinat Nagema, Radebeul, Germany) and are uncorrected. The purity of the final compounds was checked by the HPLC separation module Waters Alliance 2695 XE (Waters Corp., Milford, MA, U.S.A.). The detection wavelength 210 nm was chosen. The peaks in the chromatogram of the solvent (blank) were deducted from the peaks in the chromatogram of the sample solution. The purity of individual compounds was determined from the area peaks in the chromatogram of the sample solution. UV spectra (λ, nm) were determined on a Waters Photodiode Array Detector 2996 (Waters Corp., Milford, MA, U.S.A.) in ca 6×10^-4^ mol methanolic solution and log ε (the logarithm of molar absorption coefficient ε) was calculated for the absolute maximum λ_max_ of individual target compounds. Infrared spectra were recorded using KBr pellets on the FT-IR spectrometer Nicolet 6700 (Nicolet - Thermo Scientific, U.S.A.). All ^1^H-NMR spectra were recorded on a Bruker AM-500 (499.95 MHz for 1H) instrument (Bruker BioSpin Corp., Germany). Chemicals shifts are reported in ppm (δ) to internal Si(CH_3_)_4_, when diffused easily exchangeable signals are omitted.

### 4.2. Synthesis

*2-Methylquinazolin-4(3H)-one* (**1**): Yield 77% of a white crystalline compound; mp 245-247 °C (lit. mp 242-244 °C [62]); HPLC purity: 98.64%; UV (nm), λ_max_/log ε: 305.1/3.53; ^1^H-NMR [(CD_3_)_2_CO] δ: 2.45 (s, 3H, CH_3_), 7.44 (t, 1H, Ar-H), 7.59 (d, *J* = 8.15 Hz, 1H, Ar-H), 7.76 (t, 1H, Ar-H), 8.13 (d, *J* = 7.94 Hz, 1H, Ar-H), 11.00 (s, 1H, NH).

*3-(2-Hydroxyethyl)-2-methylquinazolin-4(3H)-one* (**2**). Yield 52% of a white crystalline compound; mp 154-156 °C; HPLC purity: 99.67%; UV (nm), λ_max_/log ε: 305.8/3.58; ^1^H-NMR (DMSO-*d*_6_) δ: 2.65 (s, 3H, CH_3_), 3.66 (q, 2H, CH_2_), 4.12 (t, 2H, CH_2_), 5.00 (t, 1H, OH), 7.46 (t, 1H, Ar-H), 7.57 (d, *J* = 8.14 Hz, 1H, Ar-H), 7.77 (t, 1H, Ar-H), 8.08 (d, *J* = 8.00 Hz, 1H, Ar-H).

#### 4.2.1. General procedures of synthesis of Compounds **3a-k**

A mixture of compound **1** (0.01 mol) and the appropriate aldehyde (0.02 mol) was mixed thoroughly and irradiated in monomode cavity of microwave reactor using pulse sequence (3×5 minutes with 30 sec. intervals) at 250 W. During irradiation, the temperature was controlled between the range 150-180 °C. After the reaction, the mixture was cooled and washed with boiling ether. The product was crystallized from acetic acid.

*2-(E)-Styrylquinazolin-4(3H)-one* (**3a**). [63] Yield 50% of a white crystalline compound; mp 253-255 °C (lit. mp 252 °C [64]); HPLC purity: 97.95%; UV (nm), λ_max_/log ε: 321.3/3.53; ^1^H-NMR (DMSO-*d*_6_) δ: 7.00 (d, *J* = 16.23 Hz, 1H, C=C-H), 7.41 (t, 1H, Ar-H), 7.42-7.49 (m, 3H, Ar-H), 7.65-7.68 (m, 3H, Ar-H), 7.80 (t, 1H, Ar-H), 7.95 (d, *J* = 16.16 Hz, 1H, C=C-H), 8.10 (d, 1H, Ar-H), 12.35 (s, 1H, N-H).

*2-[(E)-2-(2-Methoxyphenyl)vinyl]quinazolin-4(3H)-one* (**3b**). [63] Yield 76% of a white crystalline compound; mp 234-236 °C (lit. mp 234-236 °C [64]); HPLC purity: 94.04%; UV (nm), λ_max_/log ε: 343.4/3.62; ^1^H-NMR (DMSO-*d*_6_) δ: 3.90 (s, 3H, OCH_3_), 7.02 (t, 1H, Ar-H), 7.07 (d, *J* = 16.24 Hz, 1H, C=C-H), 7.11 (d, 1H, Ar-H), 7.39 (t, 1H, Ar-H), 7.45 (t, 1H, Ar-H), 7.60 (d, 1H, Ar-H), 7.67 (d, 1H, Ar-H), 7.79 (t, 1H, Ar-H), 8.09 (d, 1H, Ar-H), 8.15 (d, *J* = 16.12 Hz, 1H, C=C-H), 12.36 (s, 1H, N-H).

*2-[(E)-2-(3-Methoxyphenyl)vinyl]quinazolin-4(3H)-one* (**3c**). [63] Yield 68% of a white crystalline compound; mp 239-241 °C; HPLC purity: 96.82%; UV (nm), λ_max_/log ε: 326.4/3.58; ^1^H-NMR (DMSO-*d*_6_) δ: 3.81 (s, 3H, OCH_3_), 6.98 (d, 1H, Ar-H), 7.01 (d, *J* = 16.81 Hz, 1H, C=C-H), 7.22 (s, 1H, Ar-H), 7.23 (d, 1H, Ar-H), 7.37 (t, 1H, Ar-H), 7.47 (t, 1H, Ar-H), 7.66 (d, 1H, Ar-H), 7.80 (t, 1H, Ar-H), 7.91 (d, *J* = 16.14 Hz, 1H, C=C-H), 8.10 (d, 1H, Ar-H), 12.31 (s, 1H, NH).

*2-[(E)-2-(4-Methoxyphenyl)vinyl]quinazolin-4(3H)-one* (**3d**). [63] Yield 33% of a white crystalline compound; mp 280-281 °C (lit. mp 284-285 °C [64]); HPLC purity: 94.36%; UV (nm), λ_max_/log ε: 322.7/3.59; ^1^H-NMR (DMSO-*d*_6_) δ: 3.80 (s, 3H, OCH_3_), 6.84 (d, *J* = 16.23 Hz, 1H, C=C-H), 7.01 (d, 2H, Ar-H), 7.45 (t, 1H, Ar-H), 7.60 (d, 2H, Ar-H), 7.64 (d, 1H, Ar-H), 7.78 (t, 1H, Ar-H), 7.90 (d, *J* = 16.08 Hz, 1H, C=C-H), 8.08 (d, 1H, Ar-H), 12.25 (s, 1H, N-H).

*2-[(E)-2-(2,4-Dimethoxyphenyl)vinyl]quinazolin-4(3H)-one* (**3e**). [63] Yield 57% of a white crystalline compound; mp 228-230 °C (lit. mp 228-230 °C [64]); HPLC purity: 96.27%; UV (nm), λ_max_/log ε: 350.1/3.67; ^1^H-NMR (DMSO-*d*_6_) δ: 3.82 (s, 3H, OCH_3_), 3.90 (s, 3H, OCH_3_), 6.63 (d, 1H, Ar-H), 6.64 (s, 1H, Ar-H), 6.94 (d, *J* = 16.15 Hz, 1H, C=C-H), 7.43 (t, 1H, Ar-H), 7.53 (d, 1H, Ar-H), 7.64 (d, 1H, Ar-H), 7.77 (t, 1H, Ar-H), 8.07 (d, *J* = 15.21 Hz, 1H, C=C-H), 8.08 (d, 1H, Ar-H), 12.26 (s, 1H, N-H).

*2-[(E)-2-(3-Chlorophenyl)vinyl]quinazolin-4(3H)-one* (**3f**). [63] Yield 93% of a white crystalline compound; mp 289 °C; HPLC purity: 96.51%; UV (nm), λ_max_/log ε: 326.4/3.59; ^1^H-NMR (CDCl_3_) δ: 6.93 (d, *J* = 16.41 Hz, 1H, C=C-H), 7.39 (d, 2H, Ar-H), 7.46-7.53 (m, 2H, Ar-H), 7.64 (s, 1H, Ar-H), 7.79 (d, *J* = 16 Hz, 1H, C=C-H), 7.77-7.83 (m, 2H, Ar-H), 8.33 (d, 1H, Ar-H), 10.64 (s, 1H, NH).

*2-[(E)-2-(2-Bromophenyl)vinyl]quinazolin-4(3H)-one* (**3g**). [63] Yield 71% of a white crystalline compound; mp 279 °C; HPLC purity: 98.84%; UV (nm), λ_max_/log ε: 326.9/3.59; ^1^H-NMR (CDCl_3_) δ: 6.93 (d, *J* = 16.47 Hz, 1H, C=C-H), 7.40 (t, 1H, Ar-H), 7.50 (t, 1H, Ar-H), 7.67 (d, *J* = 7.90 Hz, 1H, Ar-H), 7.74-7.82 (m, 4H, Ar-H), 8.12 (d, *J* = 16.45 Hz, 1H, C=C-H), 8.36 (d, *J* = 7.88 Hz, 1H, Ar-H), 10.96 (s, 1H, NH).

*2-[(E)-2-(3-Bromophenyl)vinyl]quinazolin-4(3H)-one* (**3h**). [63] Yield 43% of a white crystalline compound; mp 277-279 °C; HPLC purity: 97.34%; UV (nm), λ_max_/log ε: 326.4/3.58; ^1^H-NMR (CDCl_3_) δ: 6.91 (d, *J* = 16.33 Hz, 1H, C=C-H), 7.33 (t, 1H, Ar-H), 7.50-7.56 (m, 2H, Ar-H), 7.55 (s, 1H, Ar-H), 7.73 (d, *J* = 16.49 Hz, 1H, C=C-H), 7.76-7.82 (m, 3H, Ar-H), 8.33 (d, 1H, Ar-H), 10.31 (s, 1H, NH).

*2-[(E)-2-(4-Bromophenyl)vinyl]quinazolin-4(3H)-one* (**3i**). [63] Yield 66% of a white crystalline compound; mp 332 °C; HPLC purity: 97.64%; UV (nm), λ_max_/log ε: 326.7/3.58; ^1^H-NMR (DMSO-*d*_6_) δ: 7.03 (d, 1H, C=C-H), 7.49 (t, 1H, Ar-H), 7.61 (d, 1H, Ar-H), 7.64 (d, 2H, Ar-H), 7.66 (d, 1H, Ar-H), 7.68 (d, 1H, Ar-H), 7.81 (t, 1H, Ar-H), 7.91 (d, 1H, C=C-H), 8.11 (d, 1H, Ar-H), 12.36 (s, 1H, N-H).

*2-[(E)-2-(4-Carbaldehydephenyl)vinyl]quinazolin-4(3H)-one* (**3j**). [63] Yield 35% of a white crystalline compound; mp 218 °C; HPLC purity: 96.93%;. UV (nm), λ_max_/log ε: 337.9/3.69; ^1^H-NMR (CDCl_3_) δ: 7.16 (d, *J* = 16.13 Hz, 1H, C=C-H), 7.50 (t, 1H, Ar-H), 7.69 (d, 2H, Ar-H), 7.82 (t, 1H, Ar-H), 7.87 (d, 1H, Ar-H), 7.97 (d, 1H, Ar-H), 8.00 (d, *J* = 16.21 Hz, 1H, C=C-H), 8.11 (d, 2H, Ar-H), 9.95 (s, 1H, CHO), 10.02 (s, 1H, N-H).

*2-[(E)-2-(2,3,4-Trihydroxyphenyl)vinyl]quinazolin-4(3H)-one* (**3k**). [63] Yield 60% of a brown crystalline compound; mp 300 °C (decomp.); HPLC purity: 97.67%; UV (nm), λ_max_/log ε: 365.0/3.65; ^1^H-NMR (DMSO-*d*_6_), δ: 6.39 (d, 1H, Ar-H), 6.83 (d, *J* = 15.98 Hz, 1H, C=C-H), 6.88 (d, 1H, Ar-H), 7.40 (t, 1H, Ar-H), 7.63 (d, 1H, Ar-H), 7.75 (t, 1H, Ar-H), 8.05 (d, 1H, Ar-H), 8.10 (d, *J* = 16.05 Hz, 1H, C=C-H), 8.57 (s, 1H, OH), 9.07 (s, 1H, OH), 9.68 (s, 1H, OH), 12.19 (s, 1H, NH).

#### 4.2.2. General procedures of synthesis of Compounds **4a-e**

A mixture of styrylquinazolinone derivatives **3** (0.01 mol), *N*,*N*-dimethylaniline (0.02 mol) and phosphorus oxychloride (0.015 mol) in dry benzene (50 mL) was stirred and heated under reflux for 3 h. The reaction mixture was then cooled and filtered. The filtrate was diluted with benzene (30 mL) and the solution washed with water (50 mL), twice with 20 % aqueous NaOH (50 mL) and finally twice with water. After drying with MgSO_4_, the organic solvent was evaporated and the product obtained was crystallized from heptane.

*4-Chloro-2-(E)-styrylquinazoline* (**4a**). [63] Yield 81% of an orange crystalline compound; mp 104 °C, (lit. mp 100-101 °C [65]); HPLC purity: 99.34%; UV (nm), λ_max_/log ε: 314.9/3.67; ^1^H-NMR (DMSO-*d*_6_) δ: 7.34 (d, *J* = 15.91 Hz, 1H, C=C-H), 7.41 (t, 1H, Ar-H), 7.47 (t, 2H, Ar-H), 7.77-7.80 (m, 3H, Ar-H), 8.02 (d, 1H, Ar-H), 8.06 (t, 1H, Ar-H), 8.12 (d, *J* = 16.01 Hz, 1H, C=C-H), 8.26 (d, 1H, Ar-H).

*4-Chloro-2-[(E)-2-(2-methoxyphenyl)vinyl]quinazoline* (**4b**). [63] Yield 86% of a light yellow crystalline compound; mp 153 °C; HPLC purity: 99.95%; UV (nm), λ_max_/log ε: 345.1/3.67; ^1^H-NMR (DMSO-*d*_6_) δ: 3.98 (s, 3H, OCH_3_), 7.04 (t, 1H, Ar-H), 7.12 (d, 1H, Ar-H), 7.37 (d, *J* = 16.15 Hz, 1H, C=C-H), 7.40 (t, 1H, Ar-H), 7.78 (t, 1H, Ar-H), 7.82 (d, 1H, Ar-H), 8.01 (d, 1H, Ar-H), 8.06 (t, 1H, Ar-H), 8.27 (d, 1H, Ar-H), 8.47 (d, *J* = 16.15 Hz, 1H, C=C-H).

*4-Chloro-2-[(E)-2-(3-methoxyphenyl)vinyl]quinazoline* (**4c**). [63] Yield 81% of a light yellow crystalline compound; mp 137 °C; HPLC purity: 98.62%; UV (nm), λ_max_/log ε: 342.9/3.64; ^1^H-NMR (DMSO-*d*_6_) δ: 3.90 (s, 3H, OCH_3_), 6.98 (d, 1H, Ar-H), 7.35 (d, *J* = 15.75 Hz, 1H, C=C-H), 7.35-7.39 (m, 2H, Ar-H), 7.36 (s, 1H, Ar-H), 7.80 (t, 1H, Ar-H), 8.02 (d, 1H, Ar-H), 8.06 (d, 1H, Ar-H), 8.10 (d, *J* = 15.82 Hz, 1H, C=C-H), 8.28 (d, 1H, Ar-H).

*4-Chloro-2-[(E)-2-(4-methoxyphenyl)vinyl]quinazoline* (**4d**). [63] Yield 51% of a yellow crystalline compound; mp 130-131 °C, (lit. mp 130-131 °C [65]); HPLC purity: 97.43%; UV (nm), λ_max_/log ε: 339.9/3.64; ^1^H-NMR (DMSO-*d*_6_) δ: 3.87 (s, 3H, OCH_3_), 7.03 (d, 2H, Ar-H), 7.20 (d, *J* = 15.87 Hz, 1H, C=C-H), 7.75 (d, 2H, Ar-H), 7.76 (t, 1H, Ar-H), 7.99 (d, 1H, Ar-H), 8.04 (t, 1H, Ar-H), 8.08 (d, *J* = 15.89 Hz, 1H, C=C-H), 8.25 (d, 1H, Ar-H).

*4-Chloro-2-[(E)-2-(2,4-dimethoxyphenyl)vinyl]quinazoline* (**4e**). [63] Yield 48% of a yellow crystalline compound; mp 172 °C; HPLC purity: 98.57%; UV (nm), λ_max_/log ε: 355.0/3.67; IR (KBr, cm^-1^): 2980, 2938, 1607, 1556, 1504, 1477, 1450, 1384, 1329, 959, 768, 756; ^1^H-NMR [(CD_3_)_2_CO] δ: 3.88 (s, 3H, OCH_3_), 3.98 (s, 3H, OCH_3_), 6.64 (d, 1H, Ar-H), 6.66 (s, 1H, Ar-H), 7.27 (d, *J*=16.13 Hz, 1H, C=C-H), 7.75 (d, 1H, Ar-H), 7.60 (t, 1H, Ar-H), 7.98 (d, 1H, Ar-H), 8.02 (t, 1H, Ar-H), 8.25 (d, 1H, Ar-H), 8.40 (d, *J*=16.13 Hz, 1H, C=C-H).

### 4.3. Lipophilicity HPLC determination (capacity factor k / calculated log k)

The HPLC separation module Waters Alliance 2695 XE and Waters Photodiode Array Detector 2996 (Waters Corp., Milford, MA, U.S.A.) were used. The chromatographic column Symmetry^®^ C_18_ 5 μm, 4.6 × 250 mm, Part No. WAT054275, (Waters Corp., Milford, MA, U.S.A.) was used. The HPLC separation process was monitored by Millennium32^®^ Chromatography Manager Software, Waters 2004 (Waters Corp.). The mixture of MeOH p.a. (55.0%) and H_2_O-HPLC – Mili-Q Grade (45.0%) was used as a mobile phase. The total flow of the column was 0.9 mL/min, injection 30 μL, column temperature 30 °C and sample temperature 10 °C. The detection wavelength 210 nm was chosen. The KI methanolic solution was used for the dead time (t_D_) determination. Retention times (t_R_) were measured in minutes. The capacity factors *k* were calculated using the Millennium32^®^ Chromatography Manager Software according to formula *k* = (t_R_ - t_D_)/t_D_, where t_R_ is the retention time of the solute, whereas t_D_ denotes the dead time obtained via an unretained analyte. The log *k* values, calculated from the capacity factor *k* of the individual compounds, are shown in Table 1.

### 4.4. Lipophilicity calculations

Log *P*, *i.e.,* the logarithm of the partition coefficient for *n*-octanol/water, was calculated using the programs CS ChemOffice Ultra ver. 10.0 (CambridgeSoft, Cambridge, MA, U.S.A.) and ACD/LogP ver. 1.0 (Advanced Chemistry Development Inc., Toronto, Canada). Clog *P* values (the logarithm of *n*-octanol/water partition coefficient based on established chemical interactions) were generated by means of the CS ChemOffice Ultra ver. 10.0 software. The results are shown in Table 1.

### 4.5. Study of inhibition photosynthetic electron transport (PET) in spinach chloroplasts

Chloroplasts were prepared from spinach (*Spinacia oleracea* L.) according to Masarovicova and Kralova [66]. The inhibition of photosynthetic electron transport (PET) in spinach chloroplasts was determined spectrophotometrically (Genesys 6, Thermo Scientific, U.S.A.) using an artificial electron acceptor 2,6-dichlorophenol-indophenol (DCIPP) according to Kralova *et al*. [67] and the rate of photosynthetic electron transport was monitored as a photoreduction of DCPIP. The measurements were carried out in phosphate buffer (0.02 mol/L, pH 7.2) containing sucrose (0.4 mol/L), MgCl_2_ (0.005 mol/L) and NaCl (0.015 mol/L). The chlorophyll content was 30 mg/L in these experiments and the samples were irradiated (~100 W/m^2^) from 10 cm distance with a halogen lamp (250 W) using a 4 cm water filter to prevent warming of the samples (suspension temperature 22 °C). The studied compounds were dissolved in DMSO due to their limited water solubility. The applied DMSO concentration (up to 4%) did not affect the photochemical activity in spinach chloroplasts. The inhibitory efficiency of the studied compounds was expressed by IC_50_ values, *i.e.* by molar concentration of the compounds causing 50% decrease in the oxygen evolution rate relative to the untreated control. The comparable IC_50_ value for a selective herbicide 3-(3,4-dichlorophenyl)-1,1-dimethylurea, DCMU (Diuron^®^) was about 1.9 μmol/L [68]. The results are summarized in Table 2.

### 4.6. In vitro antimycobacterial evaluation

Clinical isolates of *Mycobacterium avium* complex CIT19/06, *M. kansasii* CIT11/06, *M. absessus* CIT21/06 and strain *M. smegmatis* MC2155 were grown in Middlebrook broth (MB), supplemented with OADC supplement (Oleic, Albumin, Dextrose, Catalase, Becton Dickinson, U.K.). Identification of these isolates was performed using biochemical and molecular protocols. At log phase growth, the 10 mL culture was centrifuged at 15,000 RPM for 20 minutes using a bench top centrifuge (Model CR 4-12 Jouan Inc. U.K). Following the removal of the supernatant, the pellet was washed in fresh Middlebrook 7H9GC broth and re-suspended in 10 mL of fresh supplemented MB. The turbidity was adjusted to match McFarland standard No. 1 (3 × 10^8^ CFU) with MB broth. A further 1:20 dilution of the culture was then performed in MB broth.

The antimicrobial susceptibility of all four mycobacteria was investigated in 96 well plate format. Here, sterile deionised water (150 µL) was added to all outer-perimeter wells of the plates to minimize evaporation of the medium in the test wells during incubation. Each dilution (150 µL) was incubated with each of the mycobacterial species (150 µL). Dilutions of each compound were prepared in duplicate. For all synthesized compounds, final concentrations ranged from 300 µg/mL to 10 µg/mL. All compounds were prepared in DMSO and subsequent dilutions were made in supplemented Middlebrook broth. The plates were sealed with parafilm and were incubated at 37 °C overnight in the case of *M. smegmatis* and *M. absessus* and for five days in the case of *M. kansasii* and *M. avium* complex. Following incubation, a 10% addition of alamarBlue (AbD Serotec) was mixed into each well and readings at 570 nm and 600 nm were taken, initially for background subtraction and subsequently after 24 hour re-incubation. The background subtraction is necessary with strongly coloured compounds which may interfere with the interpretation of any colour change. In non-interfering compounds, a blue colour in the well was interpreted as an absence of growth, and a pink colour was scored as growth. The MIC was initially defined as the lowest concentration which prevented a visual colour change from blue to pink. The results are shown in Table 2.

### 4.7. In vitro antifungal susceptibility testing

The broth microdilution test [69,70] was used for the assessment of *in vitro* antifungal activity of the synthesized compounds against *Candida albicans* ATCC 44859 (CA), *Candida tropicalis* 156 (CT), *Candida krusei* ATCC 6258 (CK), *Candida glabrata* 20/I (CG), *Trichosporon asahii* 1188 (TA), *Aspergillus fumigatus* 231 (AF), *Absidia corymbifera* 272 (AC), and *Trichophyton mentagrophytes* 445 (TM). Fluconazole (FLU) was used as the standard of a clinically used antimycotic drug. The procedure was performed with twofold dilution of the compounds in RPMI 1640 (Sevapharma a.s., Prague, Czech Republic) buffered to pH 7.0 with 0.165 mol of 3-morpholino-propane-1-sulfonic acid (MOPS, Sigma, Germany). The final concentrations of the compounds ranged from 500 to 0.975 μmol/L. Drug–free controls were included. The MIC was defined as an 80% or greater (IC_80_) reduction of growth in comparison with the control. The values of MICs were determined after 24 and 48 h of static incubation at 35 °C. For *T. mentagrophytes*, the final MICs were determined after 72 and 120 h of incubation. The results are summarized in Table 3.
